# Cardioneuroablation for the treatment of reflex syncope and functional bradyarrhythmias: A Scientific Statement of the European Heart Rhythm Association (EHRA) of the ESC, the Heart Rhythm Society (HRS), the Asia Pacific Heart Rhythm Society (APHRS) and the Latin American Heart Rhythm Society (LAHRS)

**DOI:** 10.1093/europace/euae206

**Published:** 2024-07-31

**Authors:** Tolga Aksu, Michele Brignole, Leonardo Calo, Philippe Debruyne, Luigi Di Biase, Jean Claude Deharo, Alessandra Fanciulli, Artur Fedorowski, Piotr Kulakowski, Carlos Morillo, Angel Moya, Roman Piotrowski, Sebastian Stec, Richard Sutton, J Gert van Dijk, Dan Wichterle, Hung-Fat Tse, Yan Yao, Robert S Sheldon, Marmar Vaseghi, Jose C Pachon, Maurício Scanavacca, Christian Meyer, Reshma Amin, Dhiraj Gupta, Massimo Magnano, Varun Malik, Patrick Schauerte, Win-Kuang Shen, Juan Carlos Zerpa Acosta

**Affiliations:** Department of Cardiology, Yeditepe University Hospital, İçerenköy Mah. Hastahane Sok. 4,4/1 34752 Ataşehir/İstanbul, Turkey; Department of Cardiology, IRCCS Istituto Auxologico Italiano, Hospital S. Luca, Milan, Italy; Department of Cardiology, Policlinico Casilino, Rome, Italy; Department of Cardiology, Imeldaziekenhuis, 2820 Bonheiden, Belgium; Department of Cardiology, Albert Einstein College of Medicine, at Montefiore Hospital, New York, USA; Hôpitaux de Marseille, Centre Hospitalier Universitaire La Timone, Service de Cardiologie, France and Aix Marseille Université, C2VN, 13005 Marseille, France; Department of Neurology, Medical University of Innsbruck, 6020 Innsbruck, Austria; Department of Cardiology, Karolinska University Hospital, Solna, Sweden; Department of Medicine, Karolinska Institute, 171 76 Stockholm, Sweden; Department of Clinical Sciences, Lund University, 202 13 Malmö, Sweden; Centre of Postgraduate Medical Education, Department of Cardiology, Grochowski Hospital, 04-073 Warsaw, Poland; Department of Cardiac Sciences, Libin Cardiovascular Institute, Cumming School of Medicine, University of Calgary, T2N 1N4 Calgary, AB, Canada; Molecular Arrhythmias, Centro Nacional de Investigaciones Cardiovasculares (CNIC) Carlos III C. de Melchor Fernández Almagro, 3, 28029, Madrid, España; Department of Cardiology, Hospital Universitari Dexeus, 08028 Barcelona, Spain; Centre of Postgraduate Medical Education, Department of Cardiology, Grochowski Hospital, 04-073 Warsaw, Poland; Division of Electrophysiology, Cardioneuroablation, Catheter Ablation and Cardiac Stimulation, Subcarpathian Center for Cardiovascular Intervention, Sanok, Poland; Hybrid Electrophysiology and Arrhythmia Management Program, Department of Cardiac Surgery and Transplantation, National Medical Institute of the Ministry of the Interior and Administration, Warsaw, Poland; Department of Cardiology, Hammersmith Hospital Campus, National Heart & Lung Institute, Imperial College, London, UK; Department of Neurology, Leiden University Medical Centre, Albinusdreef 2, Zuid Holland, 2333 ZA Leiden, The Netherlands; Department of Cardiology, Institute for Clinical and Experimental Medicine (IKEM), 14021 Prague, Czechia; Department of Medicine, The University of Hong Kong, Pokfulam, Hong Kong SAR; Arrhythmia Center, Fuwai Hospital, National Center for Cardiovascular Diseases Chinese Academy of Medical Sciences and Peking Union Medical College, Beijing, China; Department of Cardiac Sciences, Libin Cardiovascular Institute, Cumming School of Medicine, University of Calgary, T2N 1N4 Calgary, AB, Canada; UCLA Cardiac Arrhythmia Center Division of Cardiology, Department of Medicine, University of California, Los Angeles, CA, USA; Dante Pazzanese Cardiology Institute, São Paulo Heart Hospital, São Paulo University, São Paulo, Brazil; Arrhythmia Unit-Heart Institute (Incor), University of São Paulo Medical School, Avenida Doutor Enéas Carvalho de Aguiar 44, São Paulo CEP 05403-000, SP, Brazil; Division of Cardiology/Angiology/Intensive Care, EVK Düsseldorf, Teaching Hospital, University of Düsseldorf, Kirchfeldstraße 40, 40217 Düsseldorf, Germany; Cardiovascular Department, Kings College Hospital NHS Foundation Trust, London, UK; Department of Cardiology, Liverpool Heart and Chest Hospital, Faculty of Health and Life Sciences, University of Liverpool, Liverpool, UK; Cardiology Department, Sant'Andrea Hospital, Vercelli, Italy; Centre for heart Rhythm Disorders, University of Adelaide, Adelaide, Australia; Department of Cardiology, Herzmedizin-Berlin HMB MVZ GmbH, Rudower Chaussee 9, 12489 Berlin, Germany; Interventionelle Kardiologie DGK, Herzinsuffizienz DGK, Germany; Department of Cardiovascular Diseases, Mayo Clinic, Arizona, USA; Department of Cardiology, Mayo Clinic College of Medicine, Rochester, MN, USA; Department of Cardiology, HCor Hospital do Coracao, Sao Paulo, SP, Brasil

**Keywords:** Autonomic nervous system, Atrioventricular block, Vasovagal syncope, Reflex syncope, Sinus bradycardia

## Abstract

Cardioneuroablation has emerged as a potential alternative to cardiac pacing in selected cases with vasovagal reflex syncope, extrinsic vagally induced sinus bradycardia-arrest or atrioventricular block. The technique was first introduced decades ago, and its use has risen over the past decade. However, as with any intervention, proper patient selection and technique are a prerequisite for a safe and effective use of cardioneuroablation therapy. This document aims to review and interpret available scientific evidence and provide a summary position on the topic.

## Table of contents

1. Introduction
2. Evidence review
3. Anatomy of intrinsic cardiac autonomic nervous system for cardioneuroablation
4. Pathophysiological importance of intrinsic cardiac autonomic nervous system for cardioneuroablation
 4.1. Reflex syncope
 4.2. Extrinsic vagally induced sinus bradycardia, sinus arrest or atrioventricular block
5. Patient population: preliminary evidence from cardioneuroablation in selected patients
 5.1. Characteristics of the patients who have received cardioneuroablation
  5.1.1. Patients with reflex syncope
  5.1.2. Patients with extrinsic vagally induced sinus bradycardia and atrioventricular block not correlated with syncope
6. Preprocedural diagnostic tests
 6.1. Autonomic function tests and implantable loop recorder
 6.2. Use of atropine for patient selection
7. Intra-procedural methods to identify ablation targets
 7.1. High-frequency stimulation
 7.2. Electrogram-based approach
 7.3. Anatomically guided approach
8. Ablation methods and techniques
 8.1. Right-atrial approach
  8.1.1. Unifocal, CT-guided right-sided approach
  8.1.2. Multifocal right-sided approach
  8.1.3. Similarities, differences and additional thoughts
 8.2. Bi-atrial and left-atrial approach
  8.2.1. Left-atrial approach
  8.2.2. Bi-atrial approach
9. Procedural endpoints
10. Long-term outcomes and follow up
11. Clinical outcomes of cardioneuroablation
12. Procedural and long-term risks
 12.1. Procedural risks
 12.2. Long-term risks
13. Writing committee position
14. Conclusion
Data availability

## 1. Introduction

A marked activation of the parasympathetic tone, often combined with a transient inhibition of the sympathetic system, may cause several clinical conditions such as vasovagal reflex syncope (VVS), extrinsic vagally induced sinus bradycardia-arrest or atrioventricular block (AVB).^1,2^ As originally described by Pachon and colleagues,^3^ ablation of atrial regions characterized by atrial electrograms with multiple spectral frequencies resulted in vagal denervation which was demonstrated by heart rate (HR) increase, heart rate variability (HRV) reduction and making the heart nonresponsive to atropine.

Consequently, the potential therapeutic role of elimination of these atrial regions to reduce cardiac vagal effects by endocardial radiofrequency catheter ablation for treating conditions characterized by vagal hyperactivity (functional bradyarrhythmias) was investigated by numerous groups.^4–25^ The technique was introduced by Pachon *et al*.^4^ using spectral analysis of atrial electrograms in sinus rhythm and called cardioneuroablation (CNA).

Since its introduction, CNA has shown encouraging results and the number of CNA procedures performed by physicians caring for syncope patients has substantially risen. However, some important questions including clinical indications, methodology and long-term results remain unanswered.

The sharp rise in CNA procedures in the context of a lack of large populations, randomized studies and long-term follow up necessitates a thorough appraisal of the literature to date. This scientific statement aims to critically review and interpret available evidence for patient selection, optimal ablation strategies and provide a consensus position.

## 2. Evidence review

The document was prepared by a working group composed of contributors representing the European Heart Rhythm Association (EHRA), the Heart Rhythm Society (HRS), the Asia Pacific Heart Rhythm Society (APHRS) and the Latin America Heart Rhythm Society (LAHRS). Members of the working group were asked to perform a detailed literature review and critical interpretation of the published evidence. The summary position of the writing group is provided at the end of the document.

A systematic literature search was performed in the PubMed database from its inception until 07 January 2024. The Medical Subject Headings (MeSH) terminology and keywords were related to ‘vagal denervation’, ‘autonomic denervation’, ‘autonomic modification’ ganglionated plexus modification’, ‘ganglionated plexus ablation’, ‘cardiac denervation’, ‘cardiac ablation’ and ‘cardioneuroablation’. Results of observational studies, including retrospective and prospective cohort studies, and randomized controlled trials (RCTs) presenting clinical outcomes are presented in *Tables 1–3*. Case reports and case series were excluded.

**Table 1:** Overview of selected studies and patient demographics

First author (year)	Study design	Size, *n*	Diagnosis	Age, years	Female, *n* (%)	Atropine challenge	Follow-up, months	Outcome
Pachon (2005)^4^	RO	21	VVS, SND, AVB	47.5 ± 16	4 (19.0)	+	9.2 ± 4.1	No syncope, AVB, pause
Pachon (2011)^5^	RO	43	VVS	32.9 ± 15.0	18 (41.9)	+	45.1 ± 22.0	93.1%, syncope free
Yao (2012)^6^	RO	10	VVS	50.4 ± 6.4	7 (70.0)	−	30 ± 16	100%, syncope free
Zhao (2015)^7^	RO	11	SND	45.9 ± 10.9	3 (27.2)	+	18.4 ± 6.2	All patients reported a decrease in sinus bradycardia-related symptom score
Aksu (2016)^8^	PO	22	VVS, SND, AVB	42.68 ± 14.69	10 (45.4)	+	10.9 ± 3.3	No syncope in VVS, No pause in SND, and No AVB in 6 of 7 cases
Sun (2016)^9^	RO	57	VVS	43.2 ± 13.4	35 (61.4)	−	36.4 ± 22.2	91.2%, syncope free
Qin (2017)^10^	PO^a^	62	SND	42.1 ± 7.7 vs. 58.3 ± 8.1	20 (32.2)	+	12	All patients in Group A had symptomatic improvement,
Rivarola (2017)^11^	PO	14	VVS, SND, AVB	34.0 ± 13.8	7 (50)	—	22.5 ± 11.3	71.4%, syncope or daytime bradycardia free
Debruyne (2018)^12^	PO	20	VVS, SND	41.4 ± 18.8	9 (45)	+	6	95% reduction of syncope burden
Aksu (2019)^13^	PO	20	VVS	36.0 ± 12.8	10 (50)	+	6	90%, syncope free
Hu (2019)^14^	RO	115	VVS	42.9 ± 17.9	69 (60)	−	21.4± 13.1	92.2%, syncope free
Aksu (2020)^15^	RO	51	VVS	35.5 ± 12.2	19 (37.2)	+	22.2 ± 18	94.2%, syncope free
Pachon (2020)^16^	PO	25^b^	VVS	36.3 ± 19	12 (48)	−	>24	100%, syncope free
Debruyne (2021)^17^	PO	50	VVS, SND	42.4 ± 17	21 (42)	+	12	95% reduction of syncope burden
Aksu (2021)^18^	RO	31	AVB	40.1 ± 13	13 (41.9)	+	19.3 ± 15	93.3%, AVB free
Calo (2021)^19^	PO	18	VVS	36.9 ± 11.2	10 (55.5)	−	34.1 ± 6.1	83.4%, syncope free
Mesquita (2021)^26^	PO	13	VVS, SND, AVB	49.5 ± 16 0.4	3 (23.0)	−	8.4	No patient had recurrence of symptoms or sinus arrest Recurrence of AVB in 1 of 3 patients
Aksu (2022)^20^	RO	47	VVS, SND, AVB	35.1 ± 14	19 (40.4)	+	8.0 ± 3	100%, syncope free
Xu (2022)^21^	RO	108	VVS	51.2 ± 15.3	48 (44.4)	−	8	83.7%, syncope free
Candemir (2022)^27^	PO	23	VVS, SND, AVB	40.7 ± 13.2	10 (43.5)	−	10 ± 2.9	95.6%, syncope free
Tu (2022)^28^	RO	123	VVS	42.2 ± 17.7	69 (56.0)	−	4.0 ± 1.1^c^	73.2%, syncope free
Piotrowski (2023)^22^	PRC	24^d^	VVS	38 ± 10	13 (54.1)	+	24	92%, syncope free
Stec (2023)^23^	PO	20	ND	38	8 (40)	+	18	100%, syncope free
Wileczek (2023)^24^	RO	195	VVS, SND, AVB	56.19 ± 14.3	107 (54.8)	+	30.3 ± 10.4	95%, syncope free
Rivarola (2023)^25^	PO	36	VVS, SND, AVB	33.6 ± 16.6	14 (38.9)	−	52.1 ± 35.2	83.3%, syncope free
Francia (2023)^29^	RO	60	CSS, VVS, SND, AVB	51 ± 16	23 (38.3)	+	8	88%, syncope free
Kulakowski (2023)^30^	PO	115	VVS	39 ± 13	67 (58)	+	28 (12–75)	83%, syncope free
Santos Silva (2023)^31^	PO	19	VVS	37.8 ± 12.9	6 (31.5)	−	21.0 ± 13.2	89.4%, syncope free

AVB, functional atrioventricular block; CSS, carotid sinus syndrome; ND, not defined; PO, prospective observational; PRC, prospective randomized controlled; RO, retrospective observational; SND, functional sinus node dysfunction; VVS, vasovagal syncope.

^a^Patients were divided into 2 groups based on the age. Group A (patients <50 years of age) and Group B (patients >50 years of age).

^b^The patients with paroxysmal atrial fibrillation were excluded in the table.

^c^Follow-up duration was provided as years.

^d^Only patients who underwent cardioneuroablation were included in the table.

**Table 2:** Results of head-up tilt testing before selection of cardioneuroablation

First author (year)	Type of head-up tilt testing response before cardioneuroablation (*n*, %)					
	NP	ND	Type I	Type 2A	Type 2B	Type 3
Pachon (2005)^4^			0 (0)	5 (100)		0 (0)
Pachon (2011)^5^			2 (4.7)	12 (28.6)	28 (66.7)	0 (0)
Yao (2012)^6^		10 (100)				
Aksu (2016)^8^			2 (25)	0 (0)	6 (75)	0 (0)
Sun (2016)^9^		57 (100)				
Rivarola (2017)^11^			0 (0)	4 (100)		0 (0)
Debruyne (2018)^12^		20 (100)				
Aksu (2019)^13^			4 (20)	16 (80)		0 (0)
Hu (2019)^14^		115 (100)				
Aksu (2020)^15^			5 (9.8)	0 (0)	46 (90.2)	0 (0)
Pachon (2020)1^6^	25 (100)					
Debruyne (2021)^17^			31 (100)			0 (0)
Calo (2021)^19^			4 (22.2)	4 (22.2)	10 (55.6)	0 (0)
Mesquita (2021)^26^		3 (100)				
Aksu (2022)^20^	Different criteria applied^a^					
Xu (2022)^21^			67 (62.1)	0		41 (37.9)
Candemir (2022)^27^	23 (100)					
Tu (2022)^28^	16 (13.0)^b^		67 (54.5)	32 (26.0)		8 (6.5)
Piotrowski (2023)^22^			1 (4.1)	23 (95.8)		
Stec (2023)^23^	10 (100)					
Wileczek (2023)^24^	Different criteria applied^c^					
Rivarola (2023)^25^			0 (0)	14 (100)		0 (0)
Francia (2023)^29^			48 (100)			0 (0)
Kulakowski (2023)^30^	Different criteria applied^a^					
Santos Silva (2023)^31^	Different criteria applied^a^					

NP, head-up tilt testing was not performed for patient selection; ND, no details on type of positive response defined.

^a^ECG documented (spontaneous or tilt-induced) cardioinhibitory vasovagal syncope.

^b^Head-up tilt testing was negative in 13% of patients.

^c^A mixed patient population consisting of vasovagal syncope, carotid sinus syndrome, atrioventricular block, and tachycardia-bradycardia syndrome was included in the study.

**Table 3:** Procedural details of selected studies

First author (year)	GP detection method	Guidance for mapping	Atrium	Targeted GPs	Catheter	Ablation settings
Pachon (2005)^4^	Spectral Analysis	Scopy	Both	RSGP, RIGP, LSGP, PMLGP, LIGP, CT	4 mm NI	60–70°C/15–30 s
Pachon (2011)^5^	Spectral Analysis	Ensite	Both	RSGP, RIGP, LSGP, LIGP, PMLGP, CT	8 mm NI/I	50 J/60°Co r 30 J/45°C
Yao (2012)^6^	HFS	Ensite	Left	RSGP, RIGP, LSGP, LIGP	8 mm NI	Up to 60 W and 60°C/60 s
Zhao (2015)^7^	HFS	Carto	Both	RSGP, RIGP, LSGP, LIGP, CT	I, non-CF	30–35 W/43°C/40–60 s
Aksu (2016)^8^	Spectral Analysis + HFS	Ensite	Both	RSGP, RIGP, PMLGP	I, non-CF	Up to 35 W/43°C
Sun (2016)^9^	HFS vs. Anatomical	Ensite	Left	RSGP, RIGP, LSGP, LIGP	8 mm NI	Up to 60 W/60°C/60 s
Qin (2017)^10^	Anatomical	Carto	Both	RSGP, RIGP, LSGP, LIGP, PMLGP	I, non-CF	Up to 40 W/43°C/30 s
Rivarola (2017)^11^	Anatomical	Carto/Ensite	Both	RSGP, RIGP, LSGP, LIGP, PMLGP	I, non-CF	20–35 W/50°C/30–60 s
Debruyne (2018)^12^	Anatomical	Carto	Right	RSGP	I, CF	Up to 40 W/CF > 8 g/30 s
Aksu (2019)^13^	Spectral Analysis + HFS vs. FEGM	Ensite	Both	RSGP, RIGP, LSGP, LIGP, PMLGP	I, non-CF	Up to 35 W/43°C
Hu (2019)^14^	HFS and/or Anatomical	Ensite	Left	RSGP, RIGP, LSGP, LIGP		Up to 40 W/60°C/10–30 s
Aksu (2020)^15^	FEGM	Ensite	Both	RSGP, RIGP, LSGP, LIGP	I, non-CF	Up to 35–40 W/at least 30 s
Pachon (2020)^16^	FEGM	Ensite	Both	RSGP, RIGP, LSGP, PMLGP	I, non-CF	40 W/40°C/30–120 s
Debruyne (2021)^17^	Anatomical	Carto	Right	RSGP	I, CF	Up to 40 W/CF > 8 g/90 s
Aksu (2021)^18^	FEGM	Ensite	Both	RSGP, RIGP, PMLGP, LSGP, LIGP, MTGP	I, non-CF	Up to 35–40 W/at least 30 s
Calo (2021)^19^	FEGM + Anatomical	Carto	Right	RSGP, RIGP, PMLGP	8 mm NI	50–70 W/60°C/30–60 s
Mesquita (2021)^26^	Anatomical	Carto/Ensite	Both	RSGP, RIGP, PMLGP	I, non-CF/CF	20–25 W/43°C/30 s
Aksu (2022)^20^	FEGM	Carto/Ensite	Both	RSGP, RIGP, LSGP, LIGP	I, non-CF	Up to 35–40 W/at least 30 s
Xu (2022)^21^	HFS	Carto/Ensite	Left	RSGP, RIGP, LSGP, LIGP, PMLGP	I, CF	35 W/43°C/FTI:400 gs
Candemir (2022)^27^	Anatomical	Carto/Ensite	Right	RSGP, PMLGP	I	Up to 45W/ at least 60 s
Tu (2022)^28^	HFS and Anatomical	Carto	Left	RSGP, RIGP, LSGP, LIGP	I	Up to 40 W/60°C
Piotrowski (2023)^22^	Anatomical + ICE + FEGM	Carto	Both	RSGP, PMLGP	I, CF	31–35 W/43°C/AI:400–500
Stec (2023)^23^	Anatomical	Ensite	Both	RSGP, RIGP, PMLGP, LSGP, LIGP, MTGP	I, non-CF	ND
Wileczek (2023)^24^	Anatomical	Ensite	Both	RSGP, RIGP, PMLGP, LSGP, LIGP, MTGP	I, non-CF	ND
Rivarola (2023)^25^	Anatomical	Carto/Ensite	Both	RSGP, RIGP	I, Non-CF/CF	20–30 W/48°C/30–60 sU p to 40W/CF:8–15 g/40 s or AI:500
Francia (2023)^29^	Anatomical	Ensite	Both	RSGP, RIGP, LSGP, LIGP, MTGP	I, CF	Up to 40–50 W/at least 24 s
Kulakowski (2023)^30^	ICE + Anatomical + FEGM	Carto	Both	RAGP, RIGP, PMLGP and LSGP/LIGP when needed	I, CF	Up to 35 W, AI up to 500
Santos Silva (2023)^31^	Anatomical	Carto	Both	RSGP, RIGP, LSGP, MTGP	I, CF	AI up to 500

AI, ablation index; CF, contact force; CT, crista terminalis-lateral wall of right atrium; FEGM, fractionated bipolar atrial electrogram; HFS, high-frequency stimulation; I, irrigated; ICE, intracardiac echocardiography; LIGP, posterolateral (inferior) left atrial GP; LSGP, superior left atrial GP; MTGP, Marshall tract GP; ND, not defined; NI, non-irrigated; PMLGP, posteromedial left atrial GP; RIGP, posterior (inferior) right atrial GP; RSGP, superior (anterior) right atrial GP.

## 3. Anatomy of intrinsic cardiac autonomic nervous system for cardioneuroablation

**Figure 1:**
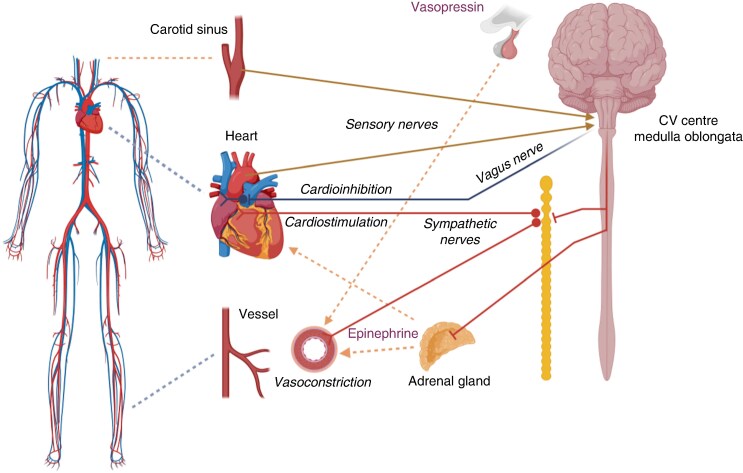
Schematization of cardiovascular regulation by the autonomic nervous system. Sensory nerves transfer signals from mechanoreceptors to cardiovascular (CV) centres in the medulla oblongata. Hypotension evokes a sympathetic response via activation of cardiac sympathetic nerves leading to tachycardia, increased inotropy and vasoconstriction of blood vessels, as well as increased release of catecholamines from the adrenal glands, and vasopressin from the hypophysis. In parallel, parasympathetic inhibition (via a reduction in the central vagal drive) contributes to tachycardia (vagal withdrawal). Conversely, reflex syncope starts with sympathetic withdrawal, vasodilation, and finally vagally mediated cardioinhibition.

The cardiac autonomic nervous system (ANS) consists of sympathetic and parasympathetic branches.^32^ Pre-ganglionic sympathetic axons originate from the spinal cord, synapse onto neurons in the cervical sympathetic chain and these post-ganglionic sympathetic neurons then innervate the myocardium (*Figure 1*).^32,33^ Pre-ganglionic parasympathetic axons of the vagus nerve originate in the brainstem in the dorsal motor nucleus and nucleus ambiguus, and synapse onto the postganglionic efferent parasympathetic neurons in the intrinsic cardiac ganglia (*Figure 1*).^33,34^ These post-ganglionic parasympathetic neurons then innervate the atrial and ventricular myocardium, the roots of caval and pulmonary veins (PVs), the sinoatrial node (SAN) and the atrioventricular node (AVN), providing cholinergic neurotransmission.^33,34^ Intrinsic cardiac ganglia cluster in anatomically well-defined areas to form ganglionated plexi (GP) that are located within the fat pads directly on the epicardium.^34^ Hence, as GPs integrate autonomic innervation to the SAN and AVN, they may serve as potential targets for parasympathetic denervation. The neurons in the GPs are widely accompanied by glia cells and may also contain convergent interneurons along with multiple afferent and efferent axons of passage and form a network on the heart. These plexus helps fine-tune autonomic reflexes, and remodel in the setting of cardiac disease.^35–37^

The afferent arm transmits signals from both atria to the brainstem. A portion of these afferents are mechanosensitive and have been localized primarily to venoatrial junctions, especially in the PV-LA junctions. They regulate blood volume and cerebral perfusion and are thought to be critical mediators of standing in humans.^38^

According to GP nomenclature of Armour *et al.*,^34^ which is commonly used to describe the distribution of GPs in experimental and clinical studies, five major atrial GPs are identified and contain most of the intrinsic cardiac ganglia. They were named based on their anatomical locations:

1. The superior (anterior) right atrial GP (RSGP), located on the anterosuperior surface of the left atrium (LA) around the ostium of the right superior PV and superolateral surface of the right atrium (RA), below the junction of the superior vena cava (SVC) and the RA.
2. The inferior (posterior) right atrial GP (RIGP), located adjacent to the interatrial groove, in proximity to the ostium of the right inferior PV.
3. The superior left atrial GP (LSGP), located on the anterosuperior surface of the LA near the ostium of the left superior PV.
4. The inferior (posterolateral) left atrial GP (LIGP), located on the inferolateral surface of the posterior LA, near the ostium of the left inferior PV.
5. The posteromedial left atrial GP (PMLGP), located on the posteromedial surface of the LA around the ostium of the coronary sinus (CS).

**Figure 2:**
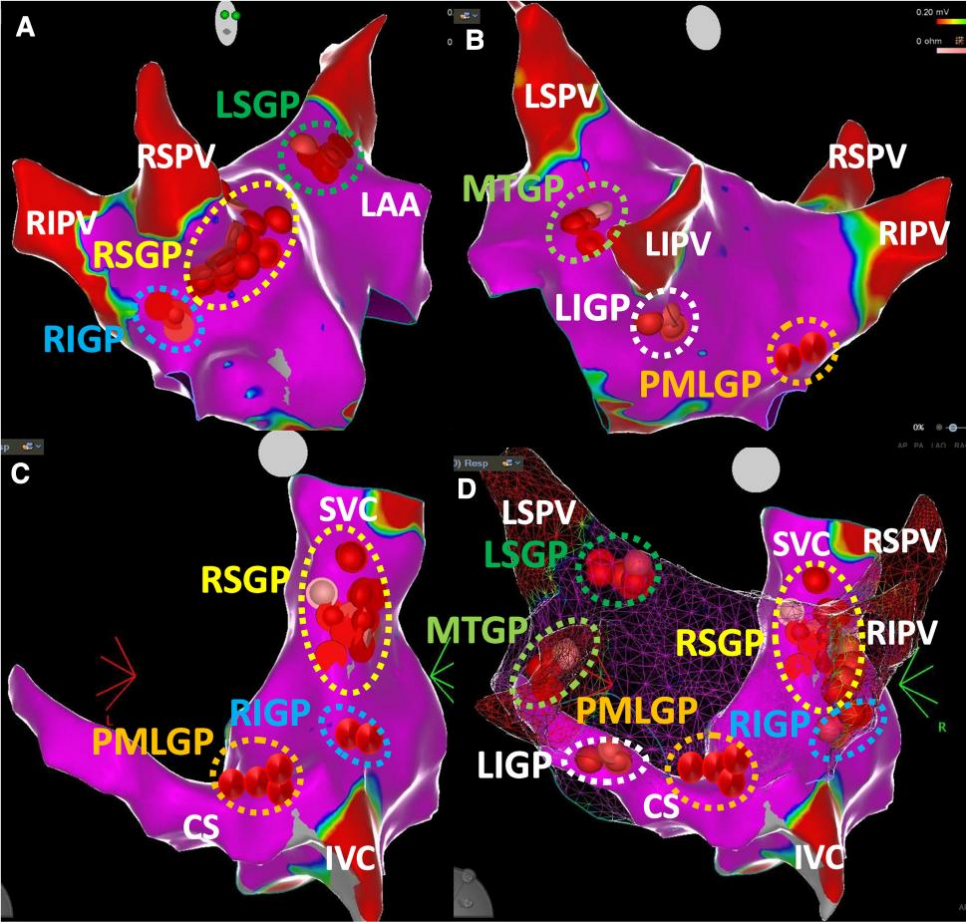
Anatomical distribution of ganglionated plexi according to the nomenclature by Armour *et al.* (Ref.^34^). *(A*) The left atrium (LA) is seen in the right anterior oblique projection. *(B*) The LA is seen in the modified posteroanterior view. *(C*) The right atrium (RA) is seen in the posteroanterior view. *(D*) Both atria are seen in the posteroanterior view. Red and pink spheres show ablation points in the relevant ganglionated plexus areas. In the LA, RSGP-RIGP can be exposed from the right anterior oblique projection, LSGP can be exposed from the anteroposterior view with cranial tilt, and LIGP-PMLGP can be exposed from the posteroanterior projection, respectively. In the RA, RSGP, RIGP, and PMLGP can be exposed from the posteroanterior projection. Please see the text for other details. *CS, coronary sinus; IVC, inferior vena cava; LAA, left atrial appendage; LIGP, inferior (posterolateral) left atrial GP LIPV, left inferior pulmonary vein; LSGP, superior left atrial GP; LSPV, left superior pulmonary vein; MTGP, the Marshall tract GP; PMLGP, posteromedial left atrial GP; RIGP, the inferior (posterior) right atrial GP; RIPV, right inferior pulmonary vein; RSGP, superior (anterior) right atrial GP; RSPV, right superior pulmonary vein; SVC, superior vena cava*.

The ligament/vein of Marshall also modulates the autonomic interactions between extrinsic and intrinsic cardiac ANS and provides parasympathetic fibres to surrounding left atrial structures and the CS (the Marshal tract GP—MTGP—neighbouring atrial myocardium to the vein of Marshall).^39^ In a recently published review article, a consensus emerged on two new terms: superior paraseptal GP for RSGP and inferior paraseptal GP for PMLGP which are frequently targeted during CNA procedures.^40^  *Figure 2* schematically illustrates the distribution of major atrial GPs which are targeted during CNA.

Based on anatomical studies, the largest number and density of autonomic ganglia which supply nerves to the SAN are typically located at the junction of the SVC and the RA (RSGP),^33,34^ and the density of these neurons increases close to the SAN.^41^ Catheter or surgical-based animal experiments indicated that the removal of intrinsic cardiac ganglia located between the inferior vena cava, proximal CS, and inferior wall of the LA eliminated the negative dromotropic effects of vagal nerve stimulation, without suppressing vagally mediated reductions in HR.^42–46^ Although successive anatomical attempts to determine location and paths of nerves that course towards the AVN have been challenging, due to the small size of these endocardial nerves,^42–44^ postganglionic nerves from the PMLGP, RIGP, and MTGP extend towards the interatrial septum and are thought to supply the AVN region.^33,39,44^

While the damage to neuronal cell bodies causes permanent effect, neurite regrowth is a well-known concept in the peripheral nervous system and leads to reinnervation of the target organ.^47^ A similar phenomenon with parasympathetic reinnervation due to axonal regeneration was demonstrated after heart transplantation, most likely in the SAN area of the allograft.^48^ Glia cells may also be involved in the regeneration process.^49^ For this reason, CNA attempts to affect the neurons located within the GPs, epicardially, rather than postganglionic nerves, reducing the possibility of reinnervation.

## 4. Pathophysiological importance of intrinsic cardiac autonomic nervous system for cardioneuroablation

### 4.1. Reflex syncope

The ANS regulates circulatory homeostasis through its direct and indirect action on the main effector organs: the heart and the vessels (*Figure 1*). Baroreceptors situated in the carotid sinus and aortic arch, as well as mechanoreceptors in the cardiac wall, provide input to the central ANS located in the medulla oblongata and are involved in maintaining the appropriate cerebral perfusion pressure.^50^ Reflex syncope describes syncope due to a specific trigger, evoking a variable combination of vasodepression and cardioinhibition, both causing a decrease in blood pressure (BP). Examples are VVS,^1^ triggered by standing, pain or fear, carotid sinus syndrome and situational syncope triggered by swallowing, defecating, urinating, coughing, sneezing, laughing and singing.^1^

The two main components of reflex syncope include vasodilation and cardioinhibition.^1^ Before common use of continuous haemodynamic monitoring, vasodilation was claimed as the main mechanism of syncope.^51^ Currently, tilt table testing, with beat-to-beat haemodynamic monitoring, is used to define the mechanism of reflex syncope.^52^ It has been observed that while cardioinhibition is often seen in younger individuals, vasodepression is more prevalent in the elderly.^53,54^ The initial vasodepressive phase^55^ is largely due to blood pooling in the abdomen and lower limbs plus a neurally mediated inhibition of the sympathetic system contributes to the reduction in venous return. Accompanying these changes observed in tilt-induced reflex syncope, there is an incomplete compensatory increase in HR relative to declining venous return in reflex-susceptible individuals.^56^ Cardioinhibition interferes at the last moment via the vagus nerve resulting in an acceleration of the fall in systemic BP due to a steep decrease in cardiac output, which leads to cerebral hypoperfusion and loss of consciousness.^56^

The trigger for cardioinhibition remains unknown, but as atropine aborts the cardioinhibitory component of the reflex, it is generally accepted that HR slowing and asystole in reflex syncope are due to cholinergic stimulation of muscarinic M2 receptors in the SAN and AVN.^40^ Moreover, it has been demonstrated that a steep increase in circulating epinephrine and vasopressin, the main compensatory mechanisms against hypotension (*Figure 2*), occurs during the onset of vasovagal reflex.^57,58^

### 4.2. Extrinsic vagally induced sinus bradycardia, sinus arrest or atrioventricular block

A small group of patients may have intermittent and frequent episodes of symptomatic sinus bradycardia, arrest or variable degree AVB, even in the absence of syncope. Episodes usually occur at rest, during sleep and with provocation testing (tilt testing and carotid sinus massage), and disappear during exercise.^2^ Despite its benign course, symptoms of fatigue, irritability, lassitude, inability to concentrate, lack of interest, forgetfulness and dizziness may be disabling in a minority of these patients. Proving a cause-effect correlation between symptoms and bradycardia is often difficult and requires prolonged electrocardiogram (ECG) monitoring (e.g. using an implantable loop recorder).^40^

These forms of bradyarrhythmia, associated with either episodes of complete loss of consciousness or pronounced hypoperfusion symptoms, are usually referred to as extrinsic or functional, to differentiate them from intrinsic (structural) SAN and atrioventricular conduction system disease.^2^ Conversely, chronotropic incompetence or persistence or worsening of AVB during exercise argue in favour of an intrinsic disturbance of sinoatrial function and atrioventricular conduction system.^2^ Epidemiology and incidence of vagally induced sinus bradycardia/asystole or AVB are not well defined and commonly researched on the basis of syncope coexistence. In a recently published nationwide study, all patients receiving their first pacemaker because of AVB before the age of 50 were investigated for aetiology. The aetiology was cardioinhibitory reflex in 5% of cases and remained unknown in 50.3% of cases.^59^

## 5. Patient population: preliminary evidence from cardioneuroablation in selected patients

### 5.1. Characteristics of the patients who have received cardioneuroablation

#### 5.1.1. Patients with reflex syncope

Although reflex syncope is benign in the vast majority of cases and often preceded by prodromal symptoms, unpredictable recurrent syncopal episodes may result in injury and impaired quality of life.^1,60^ Severe forms associated with frequent recurrences, debilitating symptoms, repeat injury and limited prodrome may warrant alternative therapy after failure of non-pharmacological and pharmacological treatment options. According to the 2021 ESC Guidelines on cardiac pacing and cardiac resynchronization therapy, dual-chamber pacing is recommended in patients >40 years of age with severe, unpredictable and recurrent syncope and a dominant cardioinhibitory response.^2^ CNA was only mentioned, in the 2018 syncope guideline as a modality that lacks strong evidence for its use in syncope.^1^ To date, in the only RCT of CNA in VVS^22^ (*Table 1*), the inclusion criteria were: (i) at least one documented episode of spontaneous reflex syncope during the preceding 12 months or one prior syncopal event leading to injury and two other pre-syncopal events; (ii) ECG of spontaneous asystolic syncope or symptomatic >3 s asystolic pause or bradycardia <40 bpm during a tilt test; and (iii) >25% increase in sinus rate after intravenous atropine injection (0.04 mg/kg for patients weighing <50 kg and with 2 mg of atropine in the remaining cases). The mean age of the 48 included patients was 38 ± 10 years. The patients had had a cumulative number of 10 ± 9 spontaneous syncopal episodes prior to study recruitment and an average of 3 ± 2 episodes in the year prior to CNA.^22^ The index event was asystolic syncope, spontaneous ECG-documented or tilt-induced, with a mean pause of 17 ± 15 s due to sinus arrest (87% of cases) or AVB (13% of cases).

In a recent metanalysis, 14 studies (1 RCT, 1 nonrandomized clinical trial and 12 observational studies) including 465 patients (mean age 39.8 ± 4.0 years; 53.5% females), who underwent CNA for VVS, were evaluated for freedom from syncope.^61^ The patients from most studies had on average ≥3 syncopal episodes in the previous year. A tilt test was performed in 94% of cases. When the Vasovagal Syncope International Study (VASIS) classification^62^ was available, the cardioinhibitory form was present in 66%, a mixed form in 31% and the vasodepressor form in 2% of cases. Thus far, patients >60 years of age have been poorly represented in CNA studies (*Table 1*). Furthermore, the results seem less satisfying as evidenced by the absence of an increase in sinus rate after ablation,^10,29^ higher syncope recurrence rate,^63^ and no improvement in quality of life in the older population.^10^ There are only case reports and a small case series with shorter follow-up for other types of reflex syncope.^10,29,63,64^

#### 5.1.2. Patients with extrinsic vagally induced sinus bradycardia and atrioventricular block not correlated with syncope

While in patients with cardioinhibitory reflex syncope, bradycardia is restricted to the time of occurrence of syncopal episodes and the rhythm is normal outside these episodes, some patients may have frequent symptomatic episodes of sinus bradycardia <40 bpm or sinoatrial block >3 s or AVB (usually second-degree Type 1 or 2:1 AVB), even in absence of syncope. Some of these patients suffer from symptoms of fatigue, irritability, lassitude, inability to concentrate, lack of interest, forgetfulness and dizziness that sometimes greatly impairs their quality of life.^2^ Nevertheless, a clear cause-effect correlation between symptoms and bradycardia is often difficult to prove in such settings. In these patients, the extrinsic (vagally induced-functional) nature of sinus bradycardia and/or conduction disturbances is suspected, because bradycardia is intermittent. Clinical features, pharmacological manoeuvres or an electrophysiological study may help differentiate extrinsic from intrinsic bradycardia. However, it must be borne in mind that a dual mechanism may frequently exist in elderly adults, namely a subtle decrease of the intrinsic properties of the SAN automaticity due to a degenerative ageing process is almost always present, even in patients with dominant extrinsic features.^65^ No controlled trial has yet been performed in patients with sinus bradycardia and/or paroxysmal AVB unrelated to syncope. There is insufficient evidence except for a few small observational studies and case reports demonstrating improvement of sinus function and atrioventricular conduction after CNA^4,8,17,18^ (*Table 1*).

However, the populations enrolled in the above studies were heterogeneous also included patients with syncope, making results difficult to interpret. The great overlap between VVS and extrinsic sinus node dysfunction (SND) was evidenced by the difficulty in classifying patients. For example, in a review of published cases, Aksu *et al.*^66^ defined as affected by ‘pure VVS’ those patients with a typical clinical history for VVS and a positive response to tilt testing without any accompanying bradyarrhythmia on prolonged ECG monitoring. They defined ‘pure SND’ as those patients with ECG documentation of intermittent or persistent bradycardia on prolonged ECG monitoring, without typical clinical history for VVS. These latter patients may have demonstrated positive tilt testing response too, if exposed to a vasovagal trigger during monitoring. Qin *et al.*^10^ studied the long-term effects of CNA on HR and quality of life in patients with symptomatic sinus bradycardia. The increase in HR was small in patients >50 years of age and there was no improvement in quality of life. Debruyne *et al.*^17^ enrolled 50 patients with syncope believed to be vagally mediated. During follow-up, after CNA, the estimated syncope recurrence rate at 1 year was around 25% both in the 31 patients who had a positive tilt test response and in the 19 patients who had documentation of sinus pauses >3 s during Holter or an implantable loop recorder. We assume that different terminology was used to define the same vasovagal episode when it was documented by tilt testing or by prolonged ECG monitoring.

In addition, Aksu *et al.*^18^ performed CNA in 31 of 241 consecutive patients (age 40 ± 13 years) presenting with symptomatic AVB. These patients had experienced at least one syncopal episode and had documented daytime second or third-degree AVB. The primary endpoint was freedom from any second or third-degree AVB on Holter monitoring. Over a mean follow-up of 19 ± 15 months, recurrent AVB episodes were observed in only 2 patients. In a recently published study, a patient who presented with high-degree AVB after third orthotropic heart transplantation due to rejection 7 months prior and who underwent CNA was followed for 3 months without occurrence of any new AVB episode which may support that primary activation of intrinsic cardiac ANS without the central nervous system might be the mechanism of vagal overactivity.^67^

Even if the severity of symptoms in most patients affected by extrinsic vagally induced sinus bradycardia and/or AVB is modest and does not require any therapy, RCTs specifically limited to extrinsic sinus bradycardia and/or AVB are warranted before CNA is offered for this subset of patients. Until such studies are available, CNA procedures should be applied investigational and limited to patients enrolled in RCTs.

## 6. Preprocedural diagnostic tests

### 6.1. Autonomic function tests and implantable loop recorder

**Figure 3:**
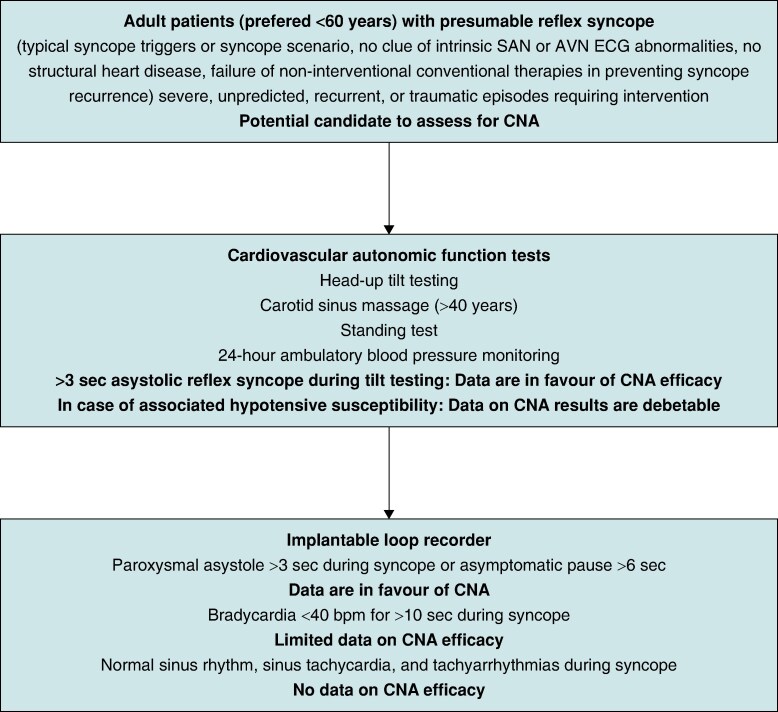
A proposed algorithm for the selection of reflex syncope patients for cardioneuroablation based on available literature. Prolonged ECG monitoring and exercise stress testing might be used to rule out intrinsic sinus or atrioventricular node disease, especially in older patients. *AVN, atrioventricular node; CNA, cardioneuroablation; SAN, sinoatrial node*.

In patients with unpredicted, recurrent or traumatic syncope episodes, the mechanism of reflex syncope should be investigated and a personalized treatment strategy should be aimed.^68,69^ According to the 2021 ESC guidelines on pacing and cardiac resynchronization therapy, in patients with ‘bradycardic phenotype’, a stepwise selection of potential candidates for pacemaker therapy should be considered. This approach was restricted to individuals older than 40 years of age, which evolved due to reluctance to implant in those younger than 40. The advent of CNA has created new therapeutic opportunities for very symptomatic younger patients.^29,69^ The so-called ‘two-step method’ involves cardiovascular autonomic function tests (CAFT) in the first stage, and long-term ECG monitoring in the second (*Figure 3*). CAFT include (at least) Valsalva manoeuvres, deep breathing and standing tests, carotid sinus massage, and tilt testing. According to the published literature, for detection of the asystolic reflex syncope mechanism, tilt-testing has been used with different protocols, so far (*Table 2*). Although there are no specific robust trials, carotid sinus massage (for patients with suspected carotid sinus syndrome >40 years) may also be used to select potential candidates by revealing a dominant mechanism.^29,69^ A tilt test result with specific relevance for CNA is an asystolic response >3 s, either abruptly or preceded by HR deceleration. Conversely, bradycardic, but non-asystolic, responses^69^ should at present not be regarded as indications for CNA, as syncope with limited bradycardia is attributable to vasodepression which is not considered amenable to parasympathetic denervation. Deep breathing and Valsalva manoeuvre are other important CAFT to be considered, in order to assess cardiovagal outflow and potential sympathetic denervation of blood vessels, and might be used to exclude baroreflex dysfunction/autonomic neuropathy as the cause of syncope.

Another important aspect of the role of CAFT and 24-hour ambulatory BP monitoring in selection of CNA candidate is the identification of the recently proposed ‘low-BP phenotype’ and hypotensive susceptibility in reflex syncope.^69–71^ This form of reflex syncope might warrant a different therapeutic approach i.e. acting on underlying BP level and unexpected BP drops, of which many patients are unaware. Addressing BP regulation issues may potentially resolve syncope and eliminate the need for intervention even if asystole has occurred. It is therefore reasonable to delay CNA to ameliorate low BP allowing reconsideration should syncope recur. More studies are needed here.

The second stage of the new proposed algorithm is the application of implantable loop recorders (*Figure 3*).^68,71,72^ In general, when the first stage, CAFT-related investigation has been negative or inconclusive, and the patient meets the overall criteria for intervention, i.e. failed guideline-recommended therapy, recurrence, severity and unpredictability of syncopal attacks, implantable loop recorders are proposed. Patients with asystolic events would qualify for potential CNA intervention, whereas non-asystolic bradycardia remains debatable.

### 6.2. Use of atropine for patient selection

Theoretically, CNA is intended to mimic the SAN and AVN effects of atropine.^5,12,18^ Therefore, to exclude patients with a degenerative disorder who would not benefit from modulation of cardiac vagal tone and to predict potential results of ablation, pre-procedure atropine response can be checked in patients who are potential candidates for CNA.^73,74^ The test should be carried out after fasting for at least 4 h with 0.04 mg/kg intravenous atropine sulphate limited to a maximum dose of 2 mg, under continuous electrocardiographic recordings for 30 min, more than 24–48 h before the procedure.

The extent of HR increase and shortening of PR interval during the pharmacological test with atropine may quantify the maximal HR and atrioventricular conduction time that could be theoretically achievable during CNA.^5,12,18^ Patients with a < 25% increase in their sinus rate or with a prolonged PR interval that is not or only partially reversible by atropine may have an intrinsic SAN or AVN disorder and should not be considered for CNA.^40^

## 7. Intra-procedural methods to identify ablation targets

### 7.1. High-frequency stimulation

High-frequency stimulation (HFS) was initially designed to identify GP location during PV isolation for atrial fibrillation (AF).^75,76^ During HFS, rapid rectangular electrical impulses with a frequency of 20 Hz, amplitude up to 20 V and pulse duration of 4–5 ms, are delivered via standard electrophysiology catheters endocardially to each GP site (Grass Stimulator, Astro Med Inc, Grass Instruments Division, West Warwick, RI, USA).^14,77,78^ The expected autonomic response can manifest in three ways: 1) vagal response (VR) characterized by immediate sinus bradycardia (increased R-R interval by 50%) and/or transient ventricular asystole (>3 s) or AVB; 2) marked shortening of the atrial refractory period near the stimulated GP; 3) and initiation of sustained AF either spontaneously or via a single atrial extra-stimulus near the GP. While the first one was used for GP mapping during CNA, remaining autonomic responses were used investigationally during AF ablation.

Schauerte *et al.*^79^ used a stimulation algorithm that allowed for selective parasympathetic nerve stimulation in the proximal CS, without simultaneous myocardial tissue stimulation. This was achieved by delivering HFS (200 Hz) within the atrial refractory period (Grass stimulator S-88, Astro-Med Inc, Grass Instruments). For this purpose, the atria were paced from the proximal CS, and the HFS trains (train duration 50 ms) were coupled to the pacing stimulus at a delay of 20 ms.

HFS may also be used as a tool to document ablation success in the targeted area. However, it should be kept in mind that a VR elicited in this way is neither sensitive nor specific to localize GPs because HFS may stimulate not only GPs but also nerves that extend from GPs towards the atrial region.^80,81^ Thus, this technique requires further evaluation. The main limitation of HFS is AF induction during mapping,^82^ requiring either electrical or pharmacological cardioversion. Another limitation is that HFS may be painful and light sedation may not be enough to ensure patient comfort.

### 7.2. Electrogram-based approach

**Figure 4:**
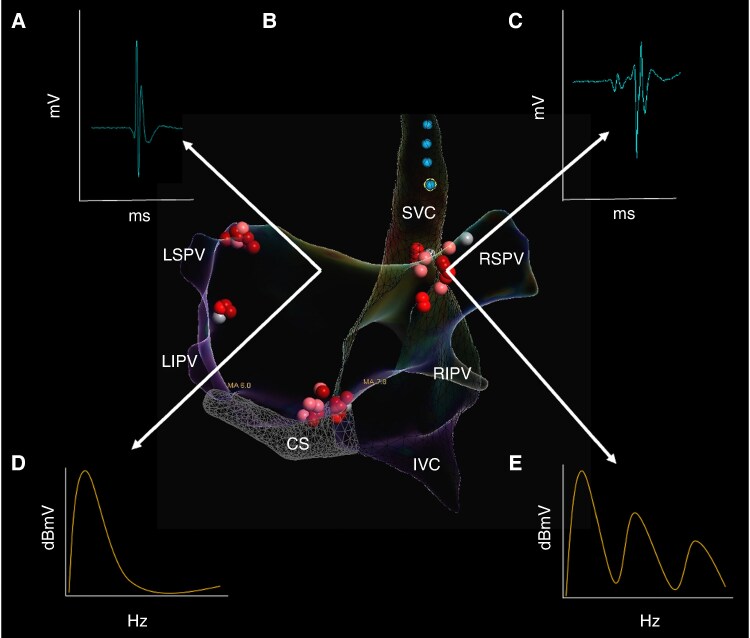
Electrogram analysis used for the identification of ganglionated plexi. Posteroanterior view of bi-atrial electroanatomical map with ablation clusters at major ganglionated plexi (GPs). *(A*) Normal bipolar atrial electrogram demonstrating two deflections. *(B*) Fractionated bipolar atrial electrogram demonstrating more than four deflections. *(C*) Frequency spectrum of compact myocardium. *(D*) Frequency spectrum of AF-nest. *CS, coronary sinus; IVC, inferior vena cava; LIPV, left inferior pulmonary vein; LSGP, superior left atrial GP; LSPV, left superior pulmonary vein; MTGP, Marshall tract GP; PMLGP, posteromedial left atrial GP; RIPV, right inferior pulmonary vein; RSGP, superior (anterior) right atrial GP; RSPV, right superior pulmonary vein; SVC, superior vena cava*.

The electrogram-based approach assumes that, in the normal heart, areas of distal endocardial inputs of GP are characterized by highly fractionated atrial electrograms, contrary to the surrounding atrial myocardium with non-fractionated or less fractionated atrial electrograms. The magnitude of fractionation can be assessed using spectral analysis^3^ or detailed visual analysis.^13^ Pachon *et al.*^4^ developed commercially available software and identified two different myocardial patterns of frequency spectra (*Table 3*). The first, with a homogeneous spectrum centred around a single frequency (approximately 40 Hz), represented the atrial myocardium located far from GP locations. The second type was characterized by multiple frequencies (>80 Hz), corresponding to the location of GPs. (*Figure 4*). Using this method, 93% of patients had no new syncope during follow-up.^5^ However, it should be remembered that any type of spectral analysis has some limitations when applied to a biological signal.^83^ Also, the reproducibility of the results of spectral analysis of so-called AF-nests has not been well established.

Time-domain analysis of fractionated bipolar atrial electrograms as a specific tool to identify GP sites was evaluated by Lellouche *et al.*^84^ Electrograms were divided based on the amplitude and number of deflections into three types: normal atrial electrograms with <4 deflections; low-amplitude (<0.7 mV) fractionated electrograms with ≥4 deflections and high-amplitude (≥0.7 mV) fractionated electrograms with ≥4 deflections. The main finding of this study was that^1^ high-amplitude fractionated electrograms are associated with ablation-induced VRs (presumable GP sites) and^2^ the corresponding prediction characteristics had a sensitivity of 72% and specificity of 91%. Recently, Aksu *et al.*^13,15,18^ used low and high-fractionated electrograms for GP mapping at filter settings between 200 and 500 Hz (*Figure 4*). These authors compared the acute procedural characteristics and syncope recurrence after fractionated electrogram-guided CNA in patients with vagally mediated bradyarrhythmias, performed by first-time operators at a single high-volume operator centre.^20^ Although the procedure time was longer in the first-time GP ablation group, acute procedural success was achieved in all cases, without any major adverse events. Over a mean follow-up duration of 8 ± 3 months (range 2–24 months), none of the patients suffered from syncope.

Time-domain analysis of fractionated electrograms also has limitations in both specificity and sensitivity. Specificity may be limited, as fractionated electrograms may represent areas of atrial fibrosis, PV potentials, or double potentials, in such areas in the RA as the slow pathway region, CS os or RA-SVC junction. Moreover, the adipose tissue surrounding the heart can infiltrate the atrial myocardium, causing heterogeneous activation resulting in the presence of fractionated electrograms.^85^ These limitations may also apply to spectral analysis approaches. In addition, the sensitivity may be limited, since effective radiofrequency applications, which cause total vagal denervation confirmed by extracardiac vagal stimulation (ECVS),^26^ can be performed at the areas with no fractionated electrograms (*Figure 4*). It is assumed that fractionated appearance of electrograms mainly caused by physical presence of GPs rather than their physiological role on innervation.

### 7.3. Anatomically guided approach

The anatomically guided approach can be applied in two different ways: as an adjunct to electrogram analysis or HFS,^4,5,7,8^ or purely anatomical, as a stand-alone strategy using isolated right-atrial^12,17^ or left-atrial/bi-atrial approaches^9,14,23–25,86^ (*Table 3*). The technique is based on empirical ablation on the presumed anatomic location of the GPs. Computed tomography (CT)-guided limited ablation on the RSGP area was developed by Debruyne *et al.*^12,17,30^ Other groups have reportedly empirically targeted 3–5 of 6 main GPs (RSGP, RIGP, LSGP, LIGP and PMLGP) via left-atrial or bi-atrial approaches.^9,14,23–25^ In a recently published study including 115 patients with VVS, intracardiac echocardiography was used to identify important anatomical landmarks and epicardial fat pads with presumed GP.^87^ CT imaging of epicardial adipose tissue (CT-EAT) might be helpful in identifying GPs that are verified by HFS.^88^ In a recently published paper, Francia *et al.*^89^ used CT-EAT with attenuation of −190 to −30 Hounsfield Units using ADAS3D software (Galgo Inc) to identify GP sites.

Although a recently published meta-analysis did not show any significant differences in freedom from syncope based on the technique used to identify GPs,^61^ perhaps in the future, more accurate methods for GP identification like direct visualization of GP using CT or positron emission tomography may become available.

## 8. Ablation methods and techniques

### 8.1. Right-atrial approach

The right-sided CNA approach is derived from anatomical studies demonstrating that a high proportion of autonomic ganglia are located in the RSGP, the RIGP, PMLGP and from the interactions between the left and right septal structures (*Table 3*).^32–34^ The right-sided ablation of GPs was first investigated in patients with vagal AF.^27^

#### 8.1.1. Unifocal, CT-guided right-sided approach

After creation of electroanatomical map of the RA and the surrounding veins, a target line at the posteroseptal side of the junction between the RA and the SVC was annotated on the CT images merged into the CARTO system.^12,17,30^ The radiofrequency applications were delivered along the line.

#### 8.1.2. Multifocal right-sided approach

A multifocal right sided approach has been suggested by several studies. After creation of electroanatomic map of the RA and the surrounding veins, empirical broad radiofrequency ablation is performed in the following right atrial regions: the superoposterior area (adjacent to the junction of the SVC and the posterior surface of the RA), the middle posterior area (posterior surface of the RA adjacent the interatrial groove), the inferoposterior area (CS ostium and near the atrioventricular groove).^19,27^ Whereas the initial approach was pure anatomical, the authors guide their cloud-like shape ablation by high-amplitude fractionated electrograms in subsequent work.^19^ In another study, authors targeted RSGP and PMLGP, purely anatomically.^28^ Notably, cloud-like ablation sets may cause atrial fibrosis in a structurally normal atrium and may facilitate the development of AF in subsequent years, especially when natural fibrosis due to ageing develops.

#### 8.1.3. Similarities, differences and additional thoughts

Although a pronounced VR was observed in 21–36% of patients when targeting the RSGP with the multifocal approach,^19,28^ the authors reported no VR during RSGP ablation with a unifocal approach.^17^ One plausible explanation is that a broader ablation set in the multifocal approach may cause axonal stimulation in the distal part of the LSGP-RSGP pathway. However, there is no data show There is no data showing clinical impact of VR during RSGP ablation ing clinical impact of VR during RSGP ablation. Although the distance between the left and the right endocardium surrounding the RSGP is only 3 ± 1 mm, any GP sandwiched between the left and right endocardium is heated by radiofrequency on one side and cooled contralaterally by the blood flow of the adjacent atrium.^12,40^ Therefore, the delivery of higher energy to targeted sites could be more important for unilateral approaches.

### 8.2. Bi-atrial and left-atrial approach

#### 8.2.1. Left-atrial approach

In the index work, after creation of the three-dimensional endocardial surface of the LA and PVs, GPs were located by HFS and ablated in the sequence of LSGP followed by LIGP, RSGP and RIGP.^6^ The endpoint of the procedure was the inhibition of the VR at each target during radiofrequency after at least 60 s of radiofrequency energy delivery. In the following work, the authors compared HFS-based GP ablation with anatomically guided GP ablation.^9^ In addition to the previously explained 4 GP sites, MTGP was integrated into the ablation protocol. Presumed GP sites were ablated sequentially from LSGP to MTGP, LIGP, RSGP and RIGP. No statistical differences were found between the HFS and anatomically guided ablation groups in either freedom from syncope or recurrent prodromes. In their latest work, authors performed left atrial ablation in LSGP, LIGP, RSGP and RIGP by using a combination of HFS anatomically guided ablation in patients with VVS.^31^

#### 8.2.2. Bi-atrial approach

After the original description of CNA by spectral mapping and fluoroscopy guidance in 2005 by Pachon and colleagues,^4^ the same group proposed an electroanatomic-mapping-guided approach.^5^ Temperature-controlled radiofrequency energy was applied to all the sites in the LA and RA having multiple spectral frequencies in atrial electrograms. Subsequently, extended empirical anatomic ablation was performed in the following regions: between the aorta and the SVC (ablated through the SVC); between the right PVs and the RA (ablated through the LA); and the inferior−posterior interatrial septum (ablated through the LA).

Aksu *et al.*^8^ adopted a combination of spectral mapping and HFS to detect GP sites. After creation of the three-dimensional geometry of RA and LA, the atrial electrograms showing a complex fractionated pattern by spectral mapping were tagged, and HFS was then delivered to these sites. The sites showing a VR during HFS were also targeted with ablation. In the subsequent work by the same group, fractionated electrograms were targeted in both atria in the following order: the LSGP, the MTGP, the LIGP, the PMLGP, the RSGP and the RIGP via the LA and RSGP via the RA. Although previous studies focused on patients with VVS, bi-atrial anatomical guided ablation with a similar ablation order was successfully used in patients with symptomatic sinus bradycardia and AVB.^10,18^ This electrogram-guided approach demonstrated a similar clinical success in comparison to the ablation strategy based on HFS and spectral mapping.^13^

Piotrowski *et al.*^22^ performed a limited bi-atrial ablation navigated by anatomical landmarks in combination with the assessment of fractionated electrograms. Both RSGP and PMLGP were ablated bi-atrially in all patients. In case of limited acceleration of sinus rhythm, these ablation clusters were extended, and LSGP and LIGP were also ablated. Other groups used a bi-atrial ablation strategy by performing empirical ablation in presumed GP areas.^23,24,29,90^ Ablation settings are provided in *Table 3*.

Although the RSGP usually has a relatively constant anatomic location which may be accessed through the RA, the interindividual PMLGP location varies widely, and hence ablation of this structure by right-atrial approach might be more challenging.^39,91–93^

In a recently published metanalysis, freedom from syncopal recurrence with right-atrial approaches was around 82% which was significantly lower than with more extensive left-atrial ablation strategies (LA ablation only (94.0%) and bi-atrial ablation (92.7%)).^61^ Similarly, in an RCT, the acute efficacy of CNA using a right-atrial vs. left-atrial approach were compared and the left-atrial approach was associated with more vagal denervation confirmed by ECVS (82% vs. 37%, *P* = 0.008).^94^ In a recently published study, while RA-based ablation of PMLGP resulted in complete AVN denervation in 8 (40%) of 20 patients, subsequent LA ablation increased the number of denervated patients to 14 (70%).^95^ Similarly, bi-atrial ablation of PMLGP resulted in 1:1 atrioventricular conduction in 12 of 15 patients with persistent AVB.^18^ However, there is still no published RCT evaluating the clinical efficacy and the learning curves of CNA with different techniques.

## 9. Procedural endpoints

The definition of endpoints is crucial for the effective implementation of CNA.

Depending on the GP mapping method used, various procedural endpoints have been defined. In studies using an electrogram-based approach, the elimination of targeted electrograms is suggested as an ablation endpoint.^7,9,18^ In the case of the HFS approach, the ablation endpoint is the elimination of positive VRs to HFS at any site showing a positive response before ablation.^5,8,13^

**Figure 5:**
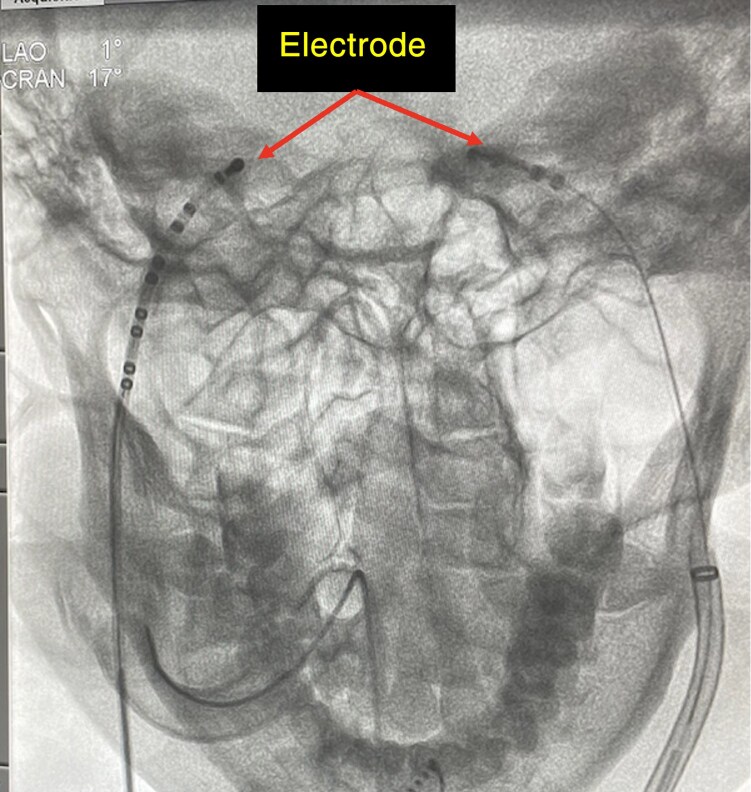
X-ray-guided extracardiac vagal stimulation. Proper position of the steerable multielectrode catheters inside the left and right internal jugular veins at the base of the skull (close to the jugular foramen) with posteromedial deflection.

Pachon *et al.*^26^ introduced a novel method to evaluate the parasympathetic effects on SAN/AVN function. By advancing a catheter within the right and/or left internal jugular vein to the jugular foramen, where the electrode is close to the vagus nerve, vagal discharge is initiated by rapid neurostimulation utilizing a neurostimulator designed by Pachon and colleagues (Sao Paulo, Brazil; pulse amplitude of 1 V/kg body weight up to 70 V, pulse width of 0.05 ms width, frequency of 50 Hz, delivered over 5 s) (*Figure 5*). The observed typical response to this ECVS is 5–10 s of sinus arrest or AVB, when simultaneous atrial pacing is delivered. ECVS can be repeated multiple times during CNA to assess the completeness of vagal denervation and selective denervation of the SAN and AVN. Elimination of this typical response may demonstrate the attainment of a high level of vagal denervation.

**Figure 6:**
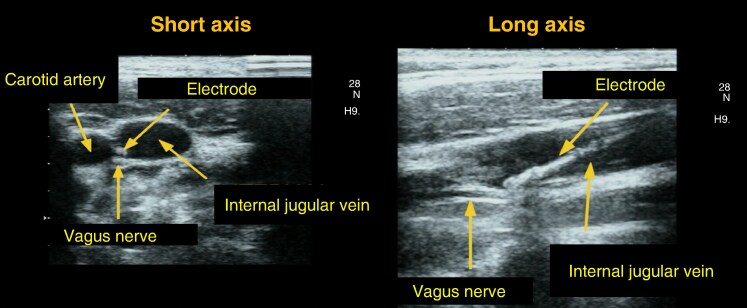
Ultrasonography-guided extracardiac vagal stimulation. The location of the pacing electrode in the vicinity of the vagus nerve is seen during ultrasonography-guided extracardiac vagal stimulation.

As a variation of the technique, Wileczek *et al.*^96^ and Piotrowski *et al.*^97^ suggested ultrasound-guided ECVS that enables the precise biplane-view positioning of the diagnostic catheter to the proximity of the vagus nerve location, in order to maximize and standardize the response to stimulation (*Figure 6*). However, this technique evaluates terminal innervation of SAN and AVN, i.e. without the possibility of differentiating the impact of neuronal and axonal lesions. It also requires general anaesthesia with myorelaxation due to the discomfort caused by stimulation.

Substantial modifications of electrophysiological parameters of the SAN (such as an increase in sinus rate or a decrease in corrected sinus node recovery time) or the AVN (including a decrease in AVN Wenckebach point, shortening of the AH interval and AVN effective refractory period) may also be used to evaluate parasympathetic denervation. However, these parameters can be influenced by factors such as sedation, pain, discomfort, stress, and the autonomic effects of the anaesthetic agents. The significant increase in sinus rate (usually >20–25%) and a shortening of the PR interval by more than 25% (if prolonged at baseline or during stable atrial stimulation), may indirectly indicate substantial vagal denervation on the SAN and AVN, respectively. At the end of the procedure, all electrophysiological parameters may be reassessed through an atropine challenge. Any substantial response to the atropine administration implies incomplete vagal denervation. A disadvantage of this test is that atropine administration prevents electrophysiological parameters from being used in further guiding the procedure.

Establishing an objective and reproducible endpoint not only aids in reducing learning curve times, but also ensures consistent results across different operators.

## 10. Long-term outcomes and follow up

The primary objective of CNA in patients with VVS and syncope associated with extrinsic vagally induced sinus bradycardia-arrest or AVB is to prevent syncopal recurrences. Therefore, the main focus of both clinical trials and clinical practice should be the assessment of syncopal recurrence, measured by time to the first recurrence or a reduction in syncope burden. To account for the possibility of late recurrence due to vagal reinnervation, follow-up should extend over several years, exceeding the typical 1–3 years duration of current studies. Recently published studies demonstrated that patients with recurrent syncope after CNA may present a vasodepressive mechanism of vasovagal reaction during control head-up tilt testing.^15,98^ However, a couple of published case reports addressed that, in patients with recurrent symptoms, the mechanism of new syncope episode and contribution of cardioinhibitory component can be revealed using carotid sinus massage, head-up tilt testing or prolonged ECG monitoring.^99,100^ In such cases, redo-procedure might be considered, after a careful discussion of all treatment options. Therefore, each recurrence of syncope requires additional clinical assessment and differential diagnosis. In addition, complex syncope (more than one aetiology of syncope e.g. co-existence of autonomic failure and orthostatic hypotension along with VVS susceptibility) may be present in a single patient. The burden of pre-syncopal episodes or bradyarrhythmia-related symptoms may be also included in clinical assessment and the patients with clinical symptoms should be re-evaluated by prolonged ECG monitoring. The reappearance of symptoms after a prolonged period of complete asymptomatic follow-up may be an indicator of re-innervation and may require close monitoring. Quality of life using a dedicated questionnaire for syncope or bradyarrhythmia may be used to follow-up patients in future clinical trials.^101^

Secondary outcome assessment is currently based on periodical resting ECG, Holter ECG and HRV evaluation. In patients with symptomatic extrinsic vagally induced sinus bradycardia-arrest and/or AVB, absence of asystole >2 s and/or second- or third-degree AVB episodes excluding non-conducted supraventricular beats during 24-h Holter recording is usually accepted as the secondary efficacy outcome of CNA. For more precise control of the CNA efficacy and occurrence of re-innervation, implantable loop recorders may be used during follow-up. Finally, Stec *et al.*^23^ implemented a control electrophysiological study and ECVS within 2–6 months after baseline CNA procedure to assess the persistence of denervation, mid-term efficacy and validation of management strategy in high-risk patients (syncope causing severe injury, syncope while driving and occupational medicine requirements).

## 11. Clinical outcomes of cardioneuroablation

Over the last 18 years, several observational and retrospective studies have shown that CNA may have salutary effects in some patients with VVS and extrinsic vagally induced SND and AVB.^4–20,86^ Selected studies on CNA are presented in *Table 1*.

The first case-control study assessed the long-term effect of CNA (51 patients) vs. conservative therapy (50 patients) for VVS with a dominant cardioinhibitory response.^102^ CNA was associated with a significantly lower risk of syncope recurrence (hazard ratio 0.23, 95% CI 0.03–0.99, *P* = 0.049) during a median follow-up of 22 months. One large meta-analysis showed 91.9% freedom from syncope after CNA, with no difference in outcomes after CNA based on various GP-mapping techniques, while more syncope recurrences were identified after right-atrial approach vs. left-atrial or bi-atrial approaches.^61^ Recently, a small RCT comparing CNA and conservative treatment recommended by ESC 2018 syncope guideline^1^ was published and showed 92% efficacy of CNA compared with 46% of optimal non-pharmacological treatment.^22^ However, the most important limitations of the existing studies are the lack of randomization with a sham-control group and relatively short follow-up, usually 2–3 years.^103^

Currently, several ongoing RCTs are comparing CNA with pacemaker implantation or sham procedure. CardNMH3 study (NCT04755101) is a multicentre, double-blind, RCT with a sham control group investigating the efficacy and safety of a CT-guided, right-sided ablation of RSGP in patients with VVS. In SAN.OK study (NCT04149886), the patients with indications for elective pacemaker implantation due to symptomatic SND will be randomly allocated in a 1:1 ratio to permanent pacemaker implantation alone or either autonomic evaluation and CNA followed by permanent pacemaker implantation if needed. In the TELE-SPACER trial (NCT05774262), the patients with indications for elective pacemaker implantation due to AVB according to 2021 ESC guidelines on cardiac pacing and positive atropine response will be randomized into two groups: pacemaker implantation or re-evaluation for pacemaker therapy after CNA.^2^ In both trials, patients without acceptance for randomization, pacemaker or CNA will be included in a standardized registry and therapy will be based on shared decision-making. The results of these trials are important to evaluate the efficacy and safety of the technique. As a non-invasive and potential alternative therapy, yoga showed a significant decrease in the number of episodes of syncope and presyncope and improvement in quality of life in two RCTs.^104,105^

## 12. Procedural and long-term risks

### 12.1. Procedural risks

Although very few complications have been reported, procedure-related major complications after CNA should not differ from those associated with established left and right atrial catheter ablation procedures. They include complications due to vascular access, cardiac perforation with haemopericardium/tamponade, large vein stenosis, injury of the phrenic nerve or coronary arteries, damage to SAN or AVN, pericarditis, oesophageal damage, bleeding and thromboembolic events. The rates of major CNA-related complications should be lower than those observed in AF ablation procedures^106^ because the radiofrequency time is lower (median 11.5 vs. 47.5 min, *P* < 0.001) during CNA as compared to AF ablation. Also, patients undergoing CNA are typically much younger with fewer co-morbidities than those undergoing AF ablation. In selected patients, mainly those with sinus nodal disorder, complications related to transseptal puncture and left atrial manipulation/ablation may be avoided by limited right-sided ablation.^12^ The damage of the SAN artery was described after bi-atrial CNA because superior septal radiofrequency lesions may be close to this artery.^107^ Although there is no published SAN artery damage after right-atrial or left-atrial CNA, the risk seems related to sites of ablation. To limit the ablation set when targeting the RSGP to the posterior aspect of the SVC facing the right superior PV antrum rather than the septal aspect can be selected to avoid this complication.

An increase in HR is an expected effect of CNA and may be symptomatic even if not fulfilling the criteria for inappropriate sinus tachycardia. In early studies, symptomatic sinus tachycardia was reported in 2–23% of individuals undergoing CNA at different follow-up periods.^5,13,25^ In a recently published paper, symptomatic sinus tachycardia was present in 27% of patients and 7% of them required long-term beta-blocker and/or ivabradine therapy.^30^ In the remaining patients, HR decreased to an asymptomatic level within 2–18 months. However, recovery of HR may predict positive response on follow-up tilt testing and possible re-innervation after CNA.^15^ Sinus tachycardia may have implications for excluding patients with heart failure with reduced or preserved ejection fraction from CNA.

### 12.2. Long-term risks

CNA results in significant attenuation of cardiac autonomic (mainly parasympathetic) regulations that manifest as increased mean HR and decreased HRV.^5,16,22,25,30^ Three studies with the longest follow-up reported a durable reduction of cardiovagal innervation lasting at least two years based on HRV variables^5,22,25^ despite signs of a statistically non-significant recovery between 12- and 18-month visits.^25^

A similar phenotype associated with increased HR and decreased HRV was described in two aetiologically distinct states which may cause adverse prognosis.

The first is cardiovascular autonomic neuropathy (CAN), mostly known from diabetes mellitus. It is a consistent, strong and independent risk factor for morbidity and mortality from all causes and cardiovascular causes.^108–111^ CAN tripled the risk of all-cause death^111^ and increased the risk of death from cardiovascular causes two- to three-fold.^110^ It doubled the rate of silent myocardial ischaemia/infarction^112^ and increased the risk of sudden cardiac death two- to ten-fold.^113^ The risks of CAN may in part be due to orthostatic hypotension and vascular dysfunction that do not apply to purely cardiovagal dysfunction after CNA. However, early CAN is predominantly cardiovagal in nature and already carries increased risks.^109,114^ Moreover, the risks were associated with HR measures like elevated HR, which are relevant for CNA.^110,114–116^

The second entity encompasses cardiac conditions causing reduced cardiovagal responsiveness. Reduced HRV and low baroreflex sensitivity predicted mortality after myocardial infarction, independently of conventional risk factors.^117^ In myocardial infarction, congestive heart failure and left ventricular dysfunction, reduced HRV was also shown to predict sudden and non-sudden cardiac death.^118^ Intact cardiovagal innervation may in turn reduce infarct size and the incidence of ventricular arrhythmias after myocardial ischaemia.^119^

Recently, in animal models, ablation of cardiac cholinergic neurons enhanced the susceptibility to ventricular arrhythmias which was likely due to suppressed cardioprotective effects of vagal innervation after CNA.^120–122^ Although a few case reports have reported potential risk for ventricular proarrhythmia after PV isolation for AF in patients without any evidence of structural heart disease attributed to the modulation of adjacent autonomic ganglia,^123,124^ a causal relationship has not been proven after 25 years of AF ablation worldwide.

There is currently no information regarding the impact of GP ablation on inter-atrial conduction and His-Purkinje system. Whether Bachmann's bundle or conduction through the CS connections from the RA to the LA are altered by bi-atrial GP ablation, remains to be studied.

## 13. Writing committee position

Well-designed randomized and sham-controlled clinical trials are needed to evaluate the clinical efficacy of CNA in highly symptomatic patients with VVS, due to the variable symptomatology, intermittent nature of symptoms, the complex pathophysiology with interindividual variability and diverse medical and interventional therapy options.

To date, there is only one small RCT and small to moderate-sized observational studies available. Accordingly, strong recommendations on the use of CNA cannot be made. Limited data report on the reduction of syncope in younger patients, with severe, unpredictable, recurrent cardioinhibitory syncope (spontaneous documented symptomatic asystolic pause(s) >3 s or asymptomatic pause(s) >6 s due to sinus arrest or AVB or >3 s asystolic syncope during tilt testing), when non-invasive conventional therapies have failed to prevent syncope recurrences. RCTs including this subgroup of patients with well-defined inclusion and exclusion criteria are important to confirm the value of CNA as an alternative therapeutic approach.

Moreover, due to the lack of a strong rationale, CNA seems to be inappropriate in the presence of a dominant vasodepressor VVS component.

For other indications, including patients with extrinsic vagally induced SND and AVB, CNA should be treated as investigational, and if considered in severely symptomatic patients, after proven failure of non-invasive conventional therapies, it requires the setting of controlled trials. Although the value of the atropine test to select patients who may benefit from CNA is not yet defined, it has been used, in the great majority of published studies, to exclude intrinsic SAN or AVN disorder in patients with SND and AVB.

Different GP mapping techniques have been suggested but comparative data are lacking and validation studies are needed. Although RSGP and PMLGP represent the main targets for parasympathetic denervation of the SAN and AVN, respectively, the bi-atrial approach may be needed in some patients to achieve full parasympathetic denervation of the AVN. ECVS might be used for intraprocedural functional assessment of selective denervation of SAN and AVN. Conventional electrophysiological indices are poor markers to determine substantial denervation of the SAN and AVN. Optimal endpoints to predict clinical outcomes are still being defined and need to be validated *in ad hoc* comparison studies.

Considering the lack of (long-term) follow-up data, it is important to follow all patients who undergo CNA, regardless of whether they are enrolled in a clinical trial or treated after shared decision-making due to pronounced symptoms, in the months after CNA with 12 lead ECGs, Holter monitoring, autonomic tests, and symptom evaluation, as appropriate. An extended follow-up (3–5 years) to define the long-term efficacy and safety of vagal denervation is desirable. The optimal follow-up technique (e.g. Holter or implanted monitoring systems, provocative tests like carotid sinus massage, head-up tilt testing, and manoeuvres for situational syncope) needs to be defined for the evaluation of symptoms, but also to determine long-term efficacy and re-innervation. Post-procedure oral anticoagulation for 1–3 months has been prescribed in patients undergoing left-atrial/bi-atrial ablation and seems reasonable until more data are available.

It is not yet known whether the post-CNA state shares the increased morbidity and mortality with other conditions associated with impaired cardiovagal innervation. Therefore, the possibility of unknown long-term risks should be discussed with patients before the procedure. Long-term studies evaluating the impact of CNA-related reduced cardiovagal responsivity on morbidity or mortality are needed to address these safety issues.

## 14. Conclusion

This interesting therapeutic modality will require more systematic and organized investigation, including randomized studies, registry-based follow-up and standardized procedural approach(es) to assess best practices and clinical outcomes.

## Data Availability

The data underlying this article are available in the article and in its online Supplementary material.
