# Correction to: Cardioneuroablation for the treatment of reflex syncope and functional bradyarrhythmias: A Scientific Statement of the European Heart Rhythm Association (EHRA) of the ESC, the Heart Rhythm Society (HRS), the Asia Pacific Heart Rhythm Society (APHRS) and the Latin American Heart Rhythm Society (LAHRS)

**DOI:** 10.1093/europace/euaf023

**Published:** 2025-02-11

This is a correction to: Tolga Aksu *et al.*, Cardioneuroablation for the treatment of reflex syncope and functional bradyarrhythmias: A Scientific Statement of the European Heart Rhythm Association (EHRA) of the ESC, the Heart Rhythm Society (HRS), the Asia Pacific Heart Rhythm Society (APHRS) and the Latin American Heart Rhythm Society (LAHRS), *EP Europace*, Volume 26, Issue 8, August 2024, https://doi.org/10.1093/europace/euae206

The version originally published omitted the following affiliation information for the author Sebastian Stec: “Hybrid Electrophysiology and Arrhythmia Management Program, Department of Cardiac Surgery and Transplantation, National Medical Institute of the Ministry of the Interior and Administration, Warsaw, Poland.”

The article has been updated to include this information.

